# Cardiac Murmur Prompting Diagnosis of Metastatic Nonseminomatous Germ Cell Testicular Neoplasia in an 18-Year-Old Patient

**DOI:** 10.1100/tsw.2005.3

**Published:** 2005-01-14

**Authors:** Steve Y. Chung, Benjamin Davies, Sheldon Bastacky, Dilip Gupta, Charles R. Pound

**Affiliations:** ^1^ Minimally Invasive Urology Institute, Cedars-Sinai Medical Center, Los Angeles, CA, USA; ^2^ Department of Urology, University of Pittsburgh Medical Center, Pittsburgh, PA, USA; ^3^ Department of Pathology, University of Pittsburgh Medical Center, Pittsburgh, PA, USA

**Keywords:** germ cell neoplasia, testis, thrombus, ventricle

## Abstract

Most retroperitoneal tumors such as renal cell carcinoma have been associated with tumor thrombus extending into the renal vein, inferior vena cava (IVC), and heart. The retroperitoneal metastatic potential of testicular tumors is well known. We report here the first instance of a cardiac murmur prompting diagnosis of metastatic testicular neoplasia in an 18-year-old patient. Chemotherapy was delayed and after successful surgical resection of the ventricular mass, the patient recovered uneventfully. This case underscores the need to pursue abnormal cardiac exams in newly diagnosed testicular cancer patients.

## CASE REPORT

An 18-year-old, previously healthy male presented to an outside hospital with a grade II/IV cardiac murmur. On physical examination, a 2- × 1-cm nontender, left scrotal mass was identified, and a scrotal ultrasound confirmed the diagnosis of a left testicular mass with a normal appearing right testis. A staging computed tomography (CT) of the head, chest, abdomen, and pelvis revealed bulky bilateral retroperitoneal and mediastinal lymphadenopathy, extensive intracaval thrombus (Fig. 1A), and bilateral lung lesions. Significant preoperative laboratory findings included serum beta human chorionic gonadotropin (β-HCG) 296 mIU/ml, alpha-fetoprotein (AFP) 1087 ng/ml, and a lactate dehydrogenase 1589 mIU/L. Radiographic studies with venacavogram and arteriogram defined the distal extent of his tumor thrombus at vertebral level L2 with cephalad extension intrahepatically and total occlusion of the left renal vein with good collateral circulation (Fig. 1B). To further evaluate his cardiac murmur, a transthoracic echocardiogram was performed and revealed a right ventricular tumor measuring 2 × 2 cm (Fig. 1C). His ejection fraction, cardiac function, and valves were within normal limits. Systemic

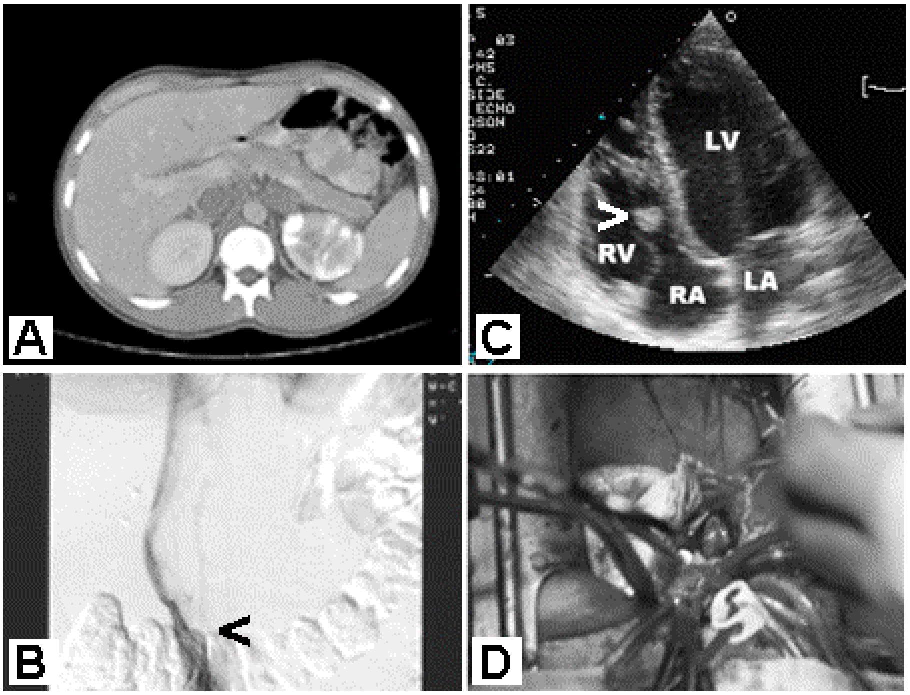

**FIGURE 1**. Tumor thrombus. (A) Contrast CT showing intracaval thrombus and hilar adenopathy. (B) Venacavagram delineating distal extent of thrombus (black arrowhead). (C) Transthoracic echocardiogram of ventricular tumor (white arrowhead). (D) Intraoperative view of ventricular tumor through open atrial chamber.

chemotherapy was delayed and surgery planned in view of the potential danger of spontaneously dislodging the ventricular tumor.

Via a median sternotomy approach and circulatory arrest, the ventricular mass was removed along with a small amount of papillary muscle (Fig. 1D). The IVC clot was also removed with aid of a fogarty catheter, and a suprarenal IVC filter was placed. A left radical orchiectomy was then performed in the standard fashion. The patient recovered uneventfully.

The left testis contained a gray-white tumor measuring 2.0 × 1.2 × 1.0 cm (Fig 2A). The tumor was comprised entirely of mature teratoma, with predominantly endodermal differentiation consistent with gastrointestinal and respiratory epithelium (Fig. 2B). The tumor was entirely confined to the testis without involvement of the tunica albuginea, rete testis, epididymis, or spermatic cord. Histology of the mass excised from the right ventricle revealed embryonal carcinoma, with no other malignant germ cell components (Fig. 2C). A panel of immunoperoxidase stains showed neoplastic cells immunoreactive for cytokeratin and CD30, but not reactive for AFP, β-HCG, and CD117 (c-kit). Furthermore, p53 immunostaining showed nuclear immunoreactivity in >50% of neoplastic cells.

Postoperatively, the patient's tumor markers normalized. He is currently undergoing adjuvant chemotherapy with cisplatin, bleomycin, and etoposide.

## DISCUSSION

Metastatic disease to the heart and pericardium is a common finding at autopsy. In one series, it was reported to be as high as 10% in patients with disseminated carcinoma of all types[1]. Patients with testicular cancer uncommonly present with tumor thrombus and rarely with cardiac involvement. In an autopsy series of only testicular cancer patients, the rate of cardiac involvement was documented at

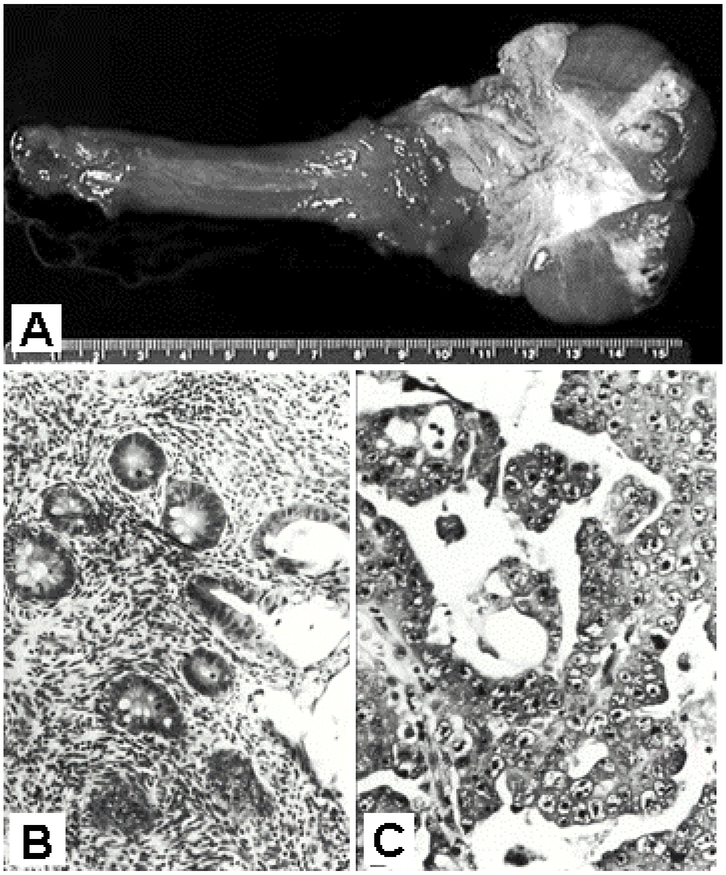

**FIGURE 2**. (A) Gross image of testicular tumor. (B) Primary tumor with mature enteric glands containing goblet cells (hematoxylin and eosin; original magnification 25×). (C) Right ventricular thrombus with high-grade carcinoma organized in trabecular and papillary formations, consistent with embryonal carcinoma (hematoxylin and eosin; original magnification 50×).

4%[2]. The majority of testicular cancer patients with cardiac metastasis have subclinical disease since they rarely manifest with overt clinical symptoms. Although there are several reports in the literature that document cardiac involvement from testicular cancer, most of these patients presented on routine follow-up postorchiectomy or had thrombus involvement only[3,4,5,6,7]. In the pediatric population, Carney's complex (sex cord stromal testicular tumors, myxomas, spotty pigmentation, and endocrine overactivity) can present initially with cardiac manifestations[8]. We report on the youngest adult patient initially presenting with a cardiac murmur due to an undiagnosed metastatic testicular cancer. Further workup of the murmur revealed a ventricular carcinoma. Chemotherapy was delayed until his cardiac mass was excised, thus highlighting the need for careful staging of all testicular cancer patients. In particular, any new abnormality in the cardiac exam warrants further testing.
